# A comprehensive computational study to explore promising natural bioactive compounds targeting glycosyltransferase MurG in *Escherichia coli* for potential drug development

**DOI:** 10.1038/s41598-024-57702-x

**Published:** 2024-03-26

**Authors:** Amneh Shtaiwi, Shafi Ullah Khan, Meriem Khedraoui, Mohd Alaraj, Abdelouahid Samadi, Samir Chtita

**Affiliations:** 1 Faculty of Pharmacy, Middle East University, Queen Alia Airport Street, Amman, P.O. Box No. 11610, Jordan; 2 Interdisciplinary Research Unit for Cancer Prevention and Treatment, Baclesse Cancer Centre, Université de Caen Normandie Inserm Anticipe UMR 1086, Normandie Univ, Research Building, F‑14000 François 3 Avenue Général Harris, BP 45026, 14076 Cedex 05 Caen, France; 3 Centre François Baclesse, Avenue Général Harris, 14076 Caen Cedex, France; 4 Laboratory of Analytical and Molecular Chemistry, Faculty of Sciences Ben M’Sik, Hassan II University of Casablanca, B. P 7955, Casablanca, Morocco; 5 Faculty of Pharmacy, University of Jerash, Jerash, Jordan; 6 Department of Chemistry, College of Science, UAEU, P.O. Box No. 15551, Al Ain, UAE

**Keywords:** MurG, Natural products, Antibacterial, Antibiotics resistance, Virtual screening, Molecular dynamics, *Escherichia coli*, Computational biology and bioinformatics, Drug discovery, Molecular biology, Chemistry

## Abstract

Peptidoglycan is a carbohydrate with a cross-linked structure that protects the cytoplasmic membrane of bacterial cells from damage. The mechanism of peptidoglycan biosynthesis involves the main synthesizing enzyme glycosyltransferase MurG, which is known as a potential target for antibiotic therapy. Many MurG inhibitors have been recognized as MurG targets, but high toxicity and drug-resistant *Escherichia coli* strains remain the most important problems for further development. In addition, the discovery of selective MurG inhibitors has been limited to the synthesis of peptidoglycan-mimicking compounds. The present study employed drug discovery, such as virtual screening using molecular docking, drug likeness ADMET proprieties predictions, and molecular dynamics (MD) simulation, to identify potential natural products (NPs) for *Escherichia coli*. We conducted a screening of 30,926 NPs from the NPASS database. Subsequently, 20 of these compounds successfully passed the potency, pharmacokinetic, ADMET screening assays, and their validation was further confirmed through molecular docking. The best three hits and the standard were chosen for further MD simulations up to 400 ns and energy calculations to investigate the stability of the NPs-MurG complexes. The analyses of MD simulations and total binding energies suggested the higher stability of NPC272174. The potential compounds can be further explored in vivo and in vitro for promising novel antibacterial drug discovery.

## Introduction

Diarrhoeagenic *Escherichia coli* (*E. coli*) pathotypes, particularly enteroaggregative *E. coli*, are one of the major food pollutants in gastrointestinal infections worldwide^1,2^. Causing more than half a million deaths and 1.7 billion morbidities of children under five yearly, thus representing an awful global health issue^3,4^. Indeed, the existence of *E. coli* has commonly been described in all countries worldwide^3,5^. And antimicrobial misuse is responsible for a frightening upsurge in bacterial resistance^6–8^. E. coli is a complex group consisting of non-pathogenic and pathogenic strains. When non-pathogenic commensals acquire additional virulence factors, juvenile, adult, pregnant, and immunocompromised individuals sometimes get diseases^9–11^. In addition, *E. coli* multi-drug resistance has been detected from numerous sources, likely to increase further with time^12,13^. Therefore, it is of utmost importance to discover a new lead with potential activity against these resistant bacteria, where the unique potential target could be the MurG glycosyltransferase^14–18^. This enzyme is crucial for synthesizing the cell wall of *E. coli* bacteria, and thus inhibitors for glycosyltransferases would be effective bactericidal agents^19–24^.

**Figure 1:**
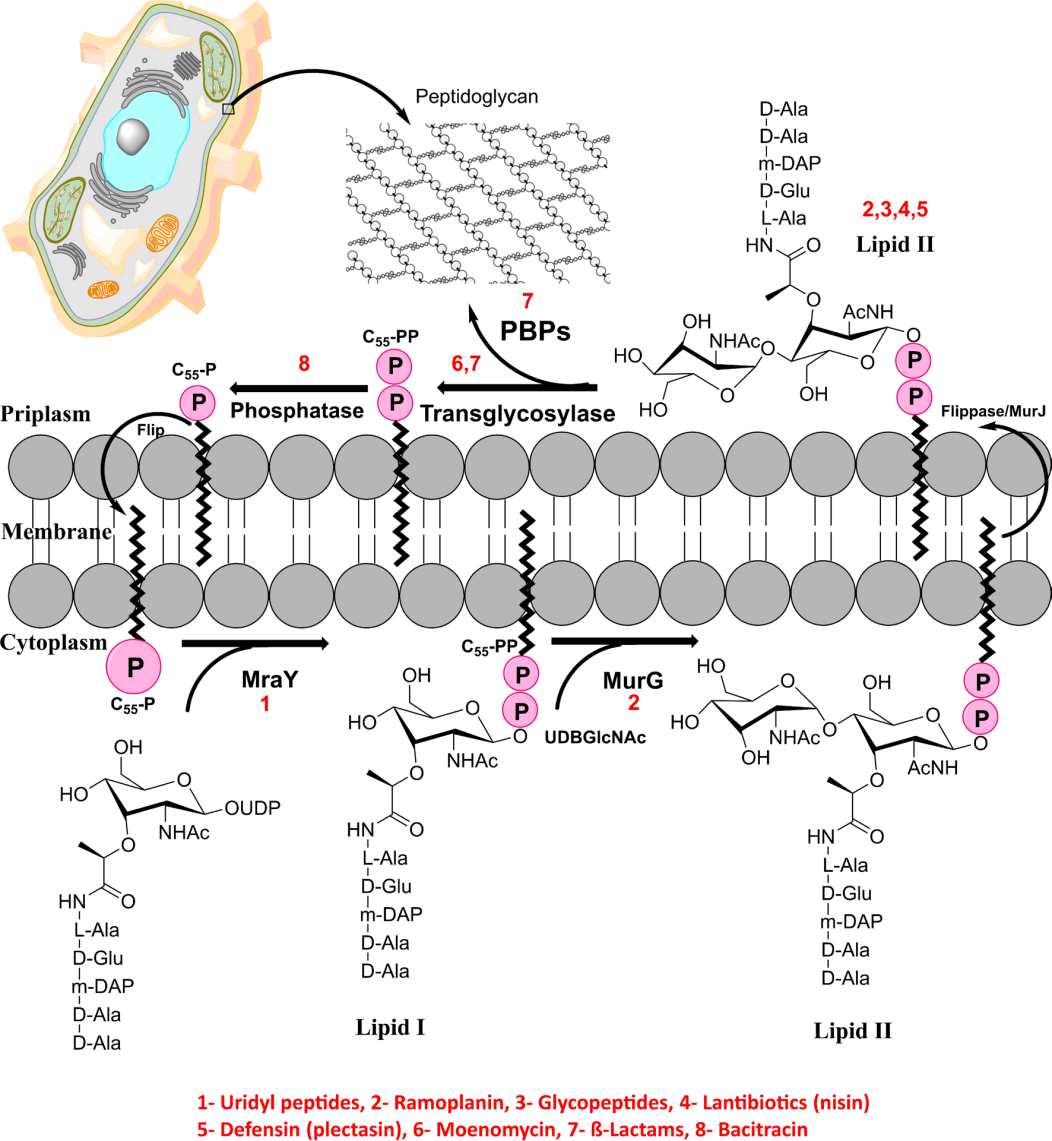
Peptidoglycan component synthesis (PCS) cycle catalyzed by MraY, MurG and penicillin-binding proteins. Numbers from (1–8) represent common antibiotics targeting the synthesis of bacterial cell walls.

Peptidoglycan constituents are produced in the cytoplasm and are relocated through the membrane for glycan polymerization via a peptidoglycan constituent synthesis (PCS) cycle that spans the plasma membrane as shown in Fig. 1^25,26^.

**Figure 2:**
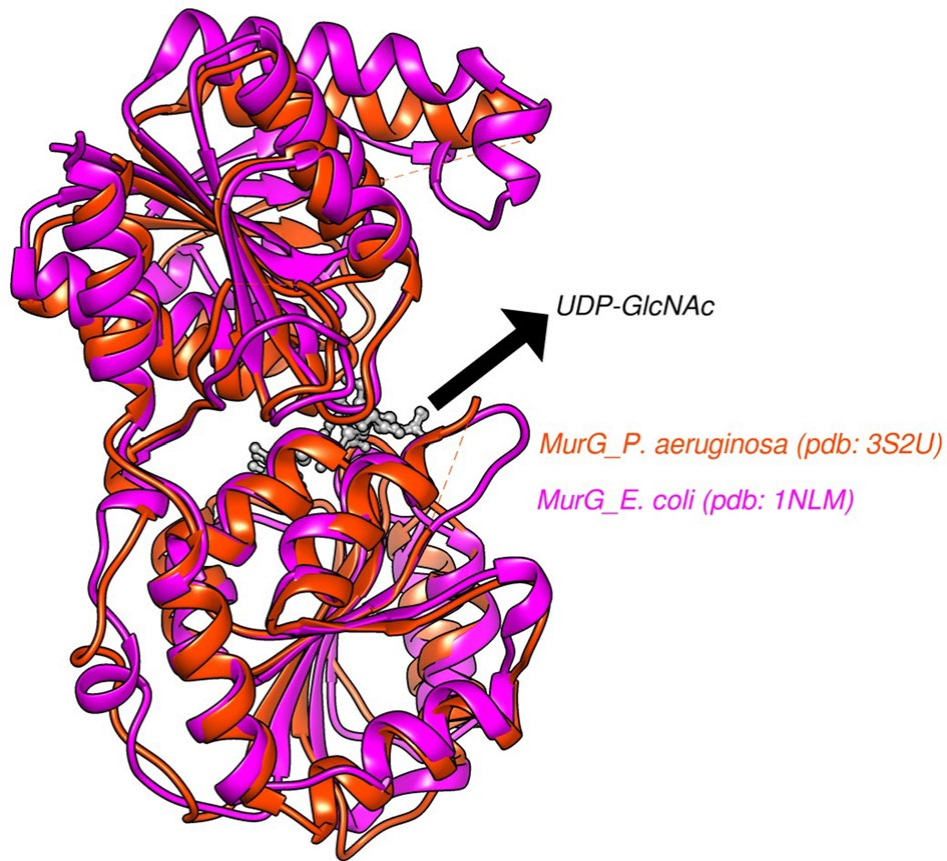
Overlays of the crystal structures of the MurG enzyme from *E. coli* (magenta, PDB code: 1nlm) and *Pseudomonas aeruginosa* (orange, pdb: 3s2u) complexed with UDP-GlcNAc (grey) in the gorge region.

MurG is an enzyme that is essential for the synthesis of peptidoglycan, a structural component of bacterial cell walls. It transfers a GlcNAc molecule from UDP-GlcNAc to lipid I to form lipid II, which is the next step in the synthesis of peptidoglycan^27,28^. The crystal structures of MurG from *E. coli* and Pseudomonas aeruginosa have been resolved, and it has been reported that both are quite similar in structure as shown in Fig. 2^29–31^. The UDP-GlcNAc molecule is bound tightly to the enzyme in the carboxy-terminal domain, and lipid I interacts with the N-terminal domain^32^. The enzymes from the two species have different conformations around the hinge domain, but the UDP-GlcNAc scaffold is bound in the same way in both enzymes^32,33^.

Several agents have been developed to inhibit glycosidase enzymes, but there are only a few uridyl peptide inhibitors available which are summarized in Table 1^34–42^. These agents are effective against MraY, an enzyme that is essential for bacterial cell wall synthesis. However, some of these agents, such as tunicamycin, are not selective and can also affect human glycol-protein synthesis^43^. Newer uridyl peptide inhibitors, such as liposidomycins and mureidomycins, are more selective for bacterial glycoprotein biosynthesis. These agents have the potential to be used as new antibiotics for the treatment of bacterial infections^34,44^.

**Table 1:** Cell wall biosynthetic antibiotics and their potential targets.

No	Antibiotics	Target	References
1	Uridyl peptides (Mureidomycin A)	MraY	^34^
2	Ramoplanin	MurG, lipid II	^35^
3	Glycopeptides	Lipid II	^36^
4	Lantibiotics (nisin)	Lipid II	^37^
5	Defensin (plectasin)	Lipid II	^38^
6	Moenomycin	Transglycosylase	^39^
7	β-Lactams	PBPs	^40,41^
8	Bacitracin	Undecaisoprenyl pyrophosphate	^42^

Many different types of antibiotics such as mannopeptimycins, lantibiotics like ramoplanin, nisin and mersacidin, and defensins like plectasin are also effective by targeting lipid II peptide, an essential component of the bacterial cell wall^35,37,38^.

Ramoplanin is a lipodepsipeptide that binds to lipid II peptide and inhibits its synthesis. This agent has broad-spectrum activity against methicillin-resistant and vancomycin-resistant gram-positive bacteria^45^. Unlike glycopeptides, ramoplanin does not bind to d-alanine-d-alanine arrangement in the cell wall precursors^36,46^.

Beta-lactam antibiotics are among the most commonly used antibiotics, and they work by inhibiting the synthesis of cell walls by interacting with penicillin-binding proteins (PBPs)^47^. PBPs are enzymes that are found in both gram-negative and gram-positive bacteria cells and play a role in peptidoglycan transpeptidase and transglycosylase action^48^. There are several mechanisms by which bacteria can develop resistance to beta-lactam antibiotics. The most common mechanism is the production of β-lactamase enzymes, which can break down the beta-lactam ring in the antibiotic molecule, rendering it ineffective^49^. Other mechanisms of resistance include alteration of enzyme PBPs, decreased diffusion of the drug to target PBPs, and efflux pumps^40,41^. There are a few antibiotics that target other steps in the cell wall synthesis pathway. Moenomycin inhibits the transglycosylase enzyme, but it is not absorbed well and is not clinically useful^39,50^. Bacitracin inhibits dephosphorylation of C55-isoprenyl pyrophosphate, but it is too toxic for widespread use^42,51^.

In summary, cell wall drugs are significant antimicrobial agents, and it is important to find new agents for this class to prevent the modification of resistance to these inhibitors. Currently, only a few methods can be used to treat infections caused by highly resistant gram-negative strains. Thus, we aimed to search novel MurG enzyme inhibitors employing a structure based virtual screening method from databases, pursued by ADMET estimation and MD simulations via in silico methods.

## Materials and methods

### Preparation of the database

The NPASS (Natural Product Activity and Species) database version 1.0 was selected to determine new inhibitors against MurG enzyme^52,53^. NPASS comprised of 35 thousand natural compounds^54^. All biological compounds were downloaded in SDF file and used for optimization for a new virtual testing process.

**Figure 3:**
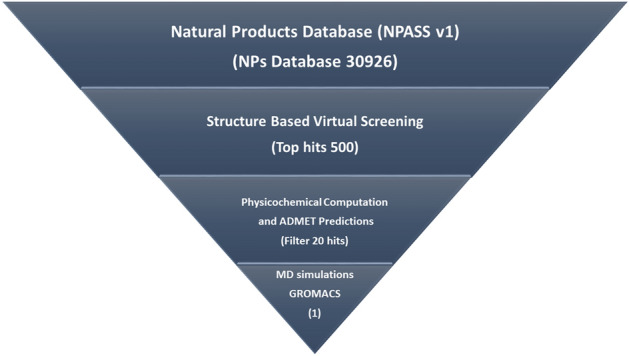
In silico screening method utilized in the study.

Figure 3 illustrates the general method used in this study. We conducted a structure based virtual screening method to choose potential MurG inhibitors from a database consisting of ~ 30,900 uncommon biological structures utilizing the NPASS version 1.0.38. The NPSS search database was selected because the compounds were extracted from 25 thousand of different natural sources. All data were determined and incorporated with ChEBI, TCMID, UNPD TCM@TaiWan, TCMSP, TM-MCTTD, HerDing, and StreptomeDB. First, biological structures from the NPASS database were saved as SDFs. OMEGA default forcefield mmff94s were then used for conformer construction and energy minimization. Finally, the best compounds with low energy structures were tested through the subsequent virtual testing methods.

NPASS compounds was designed using Discovery Studio, OpenEye, and YASARA software. The 3D optimization of the compounds was performed using Discovery Studio software, followed by energy minimization utilizing YASARA Structure^55,56^.

### Screening and docking studies

The study utilized a molecular docking method for Virtual Screening. Specifically, the bioactive compounds were prepared and subjected to molecular docking employing FRED program version3.2.0 available in OpenEye software^57–59^. Prior to docking study, the MurG enzyme was prepared utilizing the pdb2receptor program within OEDocking, and optimization was performed at a neutral pH of 7.0. Subsequently, OMEGA 2.5 was employed to form 200 conformers for each compound through standard settings^60^. The cocrystal ligand was used to define the active site within a 10 Å radius during the docking calculations. Then, the FRED default parameters were employed to calculate the binding energies of the compounds against the MurG enzyme. Ten docked conformations were generated for each ligand using the above-mentioned docking procedure. Finally, the best five hit compounds with the lowest binding energy were selected for molecular simulation study.

**Figure 4:**
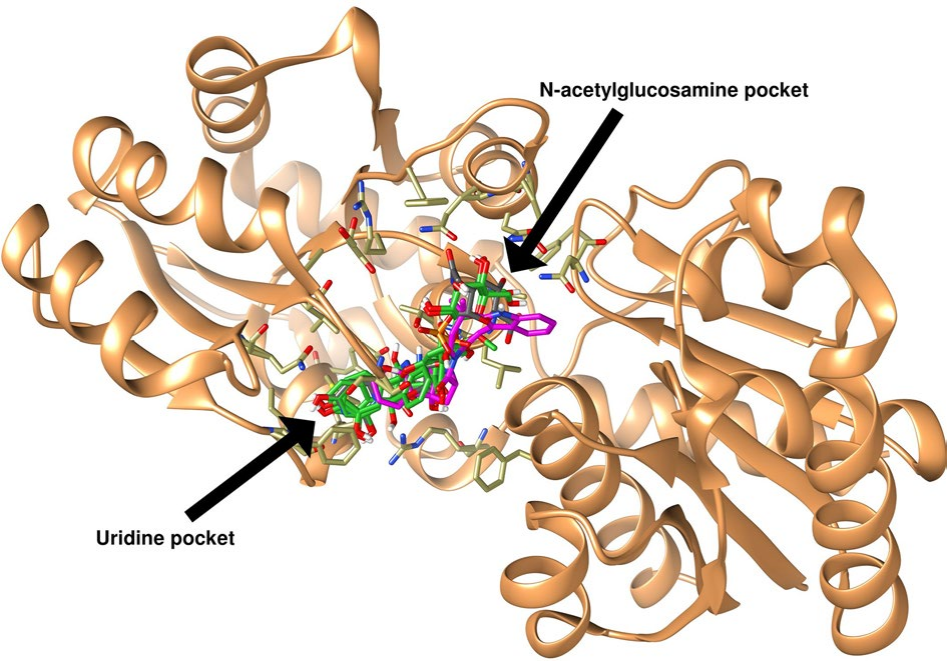
Superimposition of the UDP_GlcNAc native ligand and the top five hits interacted in the MurG active site, pdb:1nlm. Showing the binding poses in a groove between the two domains.

*E. coli* glycosyltransferase (MurG) was determined from the (pdb crystal: 1nlm) of *E. coli* K-12 MurG strain complexed to the UDP_GlcNAc. It has been observed that the UDP_GlcNAc and the natural compounds interact with the MurG catalytic site in a cleavage formed between two domains and consisting of two adjacent pockets: The N-acetylglucosamine ring is accommodated in pocket B, while the uridine forms hydrophobic interactions with pocket A, as depicted in Fig. 4. To validate the procedure, the docking was conducted utilizing the UDP_GlcNAc substrate as a reference. Additionally, for increased result reliability, the size of the docking box was expanded to encompass both the A and B pockets. The FRED docking approach was employed to optimize MurG, aiming to achieve a lower energy level. The crystal structure of UDP_GlcNAc-MurG complex, PDB ID 1nlm, was prepared for docking using Spruce v1.5.2.1 tool in OpenEye Scientific software, with default parameters^61,62^. Before running Spruce, the homodimer Chain B from protein structure was removed. This was done to isolate the active site of the protein, which is the region where the substrate, UDP_GlcNAc, binds. Spruce effectively splits existing protein–ligand complex and isolates active sites where small molecules are bound to macromolecules. Spruce performs prerequisite preparation of protein structure, including: adding hydrogen atoms to the protein structure to complete its chemical makeup. Optimizing the placement of hydrogen atoms to improve the accuracy of the structure. Expanding the asymmetric unit to its biological counterpart for the X-ray crystallography structures. This ensures that the active site is properly represented in the docked molecule. After completing these tasks, Spruce created a docking-ready receptor. The generated grid box upon the active site had a dimension of 37.884282, − 3.588333, and 20.849103 in XYZ dimensions from the obtained design units for the target UDP_GlcNAc-MurG complex^63^. Following this prerequisite step, validation of docking method was performed by redocking of co-crystalized substrate, UDP_GlcNAc and found to have an RMSD value lower than 2 Å. After though assessment of redocking and validation, all-natural products were subjected to screening. Out of the 30,926 docked natural products, they were sorted based on the binding energies of Fred ChemGuass4, and the best 500 compounds that successfully docked in the MurG binding site were chosen for the next ADMET study.

### ADMET studies

The SwissADME and admetTSAR2 was employed to calculate the drug-likeness and ADMET properties of the top docked natural product detected from the virtual screening experiment against MurG enzyme^64,65^.

### Molecular dynamics simulation

The dynamic activity of the compound with the lowest two binding energy NPs, NPC727174, NPC170742, and the fifth one NPC259098, in complex with the Glycosyltransferase enzyme MurG (pdb:1nlm), was analyzed through 300 ns simulation. The GROMACS 5.1.4 software program^66–68^ was utilized for conducting the MD simulation, employing the AMBER-FF99SB-ILDN force field^69^. The GROMACS program was employed to generate topology parameters for the MurG receptor, while the small ligands (NPC727174, NPC170742, NPC259098 and the standard UDP_GlcNAc topology parameters were created using ACPYPE from the Amber-Tools package^70^. The systems were placed to periodic cubic box spacing distance of 1.2 nm around the surface and solvated using the TIP3P solvation process at 295 K. To neutralize the systems, counter ions were added to balance the charge of the enzyme. Structural minimization was carried out for 15 thousand steps using the steepest descent protocol under consistent pressure and 295 K, followed by the Berendsen thermostat equilibration run in the NVT (constant number of particles, temperature and volume) ensemble for 200 ps at 300 K. Then, the production runs were performed using the Parrinello–Rahman barostat in the NPT ensemble (constant number of particles, temperature and pressure) for 1 ns at 1 bar and 300 K^71^. After the temperature and pressure adjustments, MD simulation runs were performed for the four different systems for 100 ns and a total of 400 ns. VDW and Coulomb interaction cutoffs were adjusted to 12.0 Å and corrected every 2 fs. Moreover, the particle mesh Ewald (PME) protocol was applied to correct ionic contacts^72^. Bond constraints with hydrogen atoms were maintained using LINCS algorithm^73^. The simulations were conducted with time step of 2.0 femtosecond, and coordinates were recorded at intervals of every 500 step. MD simulation results were analyzed by RMSD, RMSF, structure stability, transition path analysis, and free energy calculations. The visualizations were produced using PyMOL, Discovery Studio and Chimera programs^55,74,75^.

### Free energy calculations

Free energy calculations were performed using the molecular mechanics Poisson–Boltzmann surface area (MM-PBSA) method of the GROMACS software prepared using the gmx_mmpbsa tool^76^. In this study, the last 20 ns of the simulations trajectories of the standard UDP_GlcNAc, and three NPs from the top five structures (NPC727174, NPC170742, and NPC259098) complexes were chosen for energy analysis. MM-PBSA was applied to predict the average binding free energies using a Python script, MmPbSaStat.py. Moreover, the output file summary_energy.dat was obtained and contains the total binding energy of all energetic components (ΔG_Total_) including the polar solvation energy, ΔE_PSE_, solvent-accessible surface area (SASA), ΔES_SASA_, electrostatic interaction, (ΔE_ele_), and van der Waals interaction, (ΔE_vdW_). On the other hand, to calculate the average contribution of the residues to the binding energy, the Python script MmPbSaDecomp.py was used, and the results, including the binding energy for each residue, were plotted to show the energy contribution of each significant amino acid residue with its energy. The "Supplementary Python scripts S1 and S2" provide details on the average binding energy calculation and contribution of residues to the binding energy, respectively.

## Results and discussion

### Binding interactions MurG receptor

The free binding energies of the top 20 MurG-selective natural products were determined and organized based on their ChemGuass4 scores. The FRED binding results were calculated in kcal/mol and presented in (Table 2). In this study, the ChemGuass4 scores exhibit a consistent range for the binding free energies of the 20 NPs to MurG, falling within − 9.70 ± 0.50 kcal/mol, the compound NPC272174 inhibitor (− 10.23 kcal/mol) displays best binding affinity, while compound NPC154741 (− 9.12 kcal/mol) exhibits the lowest affinity. Later, the potential 5 natural compounds which showed the best ChemGuass4 score are considered for the further studies. The chemical structure, source name and ChemGuass4 score are shown in Table 3. Conversely, Table 4 illustrates the significant binding interactions of the best five compounds with the active site amino acids of MurG enzyme. Additionally, Supplementary (Table S1) provides details on the H-bond donor and acceptor pairs for the best NPs, along with their corresponding lengths.

**Table 2:** FRED binding energies of the top twenty natural compounds used in this study.

No	Name	FRED Chemgauss4 score
1	NPC272174	− 10.2568
2	NPC170742	− 10.0554
3	NPC117260	− 9.96509
4	NPC277205	− 9.96279
5	NPC259098	− 9.81826
6	NPC148409	− 9.8013
7	NPC45400	− 9.74282
8	NPC245014	− 9.62971
9	NPC265454	− 9.58556
10	NPC252590	− 9.55989
11	NPC307938	− 9.53453
12	NPC308931	− 9.4984
13	NPC214729	− 9.43665
14	NPC124300	− 9.41008
15	NPC314573	− 9.37501
16	NPC119767	− 9.36645
17	NPC263940	− 9.26997
18	NPC18185	− 9.19354
19	NPC298778	− 9.18163
20	NPC154741	− 9.12232
Standard	UDP_GlcNAc	− 6.29873

**Table 3:** Detailed information of the top five natural compounds identified used in this study.

N^o^	Code	Chemical structure	Common name	Sources	References
1	NPC272174		Okaramine I	Aspergillus aculeatus	^82^
2	NPC170742		Alpha, Beta,3,4,5,2′,4′,6′-Octahydroxydihydrochalcone	Sapium haematospermum	^83^
3	NPC117260		Patulitrin	Artemisia annua L	^84^
4	NPC277205		Quercimeritrin	Hyrtios erecta	^85^
5	NPC259098		Aflaquinolone F	Aspergillaceae	^86^

**Table 4:** Binding interaction types between the amino acid residues in the MurG active site and the best five natural products.

NPs	Interactions	Amino acids
NPC272174	H- Bond	ARG164, SER192, THR266, GLU269
	Pi-Donor H-Bond	ASN128
	Pi–Pi T-shaped	HIS19
	Amide-Pi Stacked	GLY191, SER192
	Pi-Alkyl	ALA264
NPC170742	Hydrogen Bond	ARG164, SER192, THR266, ILE245, SER192, GLU269
	Pi-S	MET248
	Pi–Pi stacked	PHE244
	Pi-Alkyl	LEU265
NPC117260	H-Bond	SER192, ALA264, GLN288, GLU269, GLN289
	Pi-Donor Hydrogen Bond	THR266, THR266
	Pi-S	MET248
	Pi–Pi stacked	PHE244
	Pi-Alkyl	LEU265
NPC277205	H-bond	SER192, ALA264, GLU269, GLN289
	Pi-donor Hydrogen bond	THR266
	Pi–Pi stacked	PHE244, MET248, LEU265
NPC259098	Hydrogen Bond	ARG164, THR266, GLU269
	Pi–Pi Stacked	PHE244
	Pi-Alkyl	ILE245, ILE245, MET248

### Drug-likeness and physicochemical analysis

ADMET study, absorption-distribution-metabolism-excretion-toxicity, was employed on the 500 natural products to estimate their Drug-likeness properties. This involved the assessment of their physicochemical properties by evaluating Lipinski, Ghose, Veber, and Egan rule violations^77,78^. According to ADMET criteria, favorable oral bioavailability is indicated by logP less than five, rotatable bonds less than ten, molecular weight less than 500, and TPSA less than 100, while good intestinal bioavailability is suggested by the number of H-bond donors less than five and acceptors less than ten. The SwissADME tool^64^ was utilized for in-depth analysis of these predictions, and the summarized results are presented in Table 5. Notably, 20 hits from the virtual screening results fell within the acceptable range of drug-like properties based on the Lipinski, Egan, Veber, and Ghose rules. Additionally, the in-silico assessment of these hits based on their physicochemical values indicated favorable pharmacokinetic properties^79^. All parameters are such as Molecular weight = (161–494 g/mol), Flexibility = (0–6) lie within the acceptable range. The number of hydrogen bond acceptors and donors measures the compound’s hydrophilicity. A greater value represents increased hydrophilicity, which leads to poor penetration and absorption. On the other hand, the number of hydrogen bond donors less than five and hydrogen bond acceptors less than ten suggests the higher penetration which would improve the absorption. All the compounds except NPC117260 lie within the acceptable number of hydrogen bonds. The (TPSA) Topological Polar Surface Area is used to measure the polar atom’s surface area for the compounds. Poorly absorbed compounds and limited cell membrane permeability have been identified as those with a TPSA > 140 Å^2^, while orally rout of administration drugs that undergo transcellular transport should generally not exceed a TPSA of about 140 Å^2^. Therefore, a lower TPSA is considered advantageous for drugs intended for oral administration that undergo transcellular transport^80^. Additionally, a compound with stronger CNS penetration is associated with toxicity and predicted to have a lower TPSA value^81^. The studied compounds show values in the range of (53–219) and four of them show high TPSA value greater than 140 Å^2^, NPC298778, NPC277205, NPC170742, and NPC117260 are considered to be poorly absorbed compounds and unable to penetrate the CNS, whereas those with TPSA values less than 140 Å^2^ are considered to increase the membrane permeability.

**Table 5:** Drug likeness analysis and physicochemical results of the best 20 natural products.

Compound	Molecular weight	Rotatable bonds	H-bond acceptors	H-bond donors	TPSA	Violations			
						Lipinski	Ghose	Veber	Egan
NPC105415	382.45	5	5	3	86.99	0	0	0	0
NPC117260	494.40	5	13	5	219.74	1	1	1	1
NPC119767	390.34	4	7	3	121.13	0	0	0	0
NPC124300	400.38	4	9	3	131.23	0	0	0	0
NPC148409	271.22	0	6	3	92.04	0	0	0	0
NPC170742	338.27	4	9	8	178.91	1	0	1	1
NPC214729	328.32	4	6	3	96.22	0	0	0	0
NPC223136	302.32	5	5	3	79.15	0	0	0	0
NPC252590	161.16	0	3	2	53.35	0	1	0	0
NPC259098	255.27	1	4	3	73.05	0	0	0	0
NPC265454	330.37	8	5	4	97.99	0	0	0	0
NPC272174	452.50	0	3	3	88.67	0	1	0	0
NPC276930	260.20	0	6	4	111.13	0	0	0	0
NPC277205	464.38	4	10	4	210.51	1	1	1	1
NPC298778	486.47	6	10	3	148.82	0	1	1	1
NPC308931	328.37	2	2	3	73.04	0	0	0	0
NPC314573	348.44	5	4	2	66.24	0	0	0	0
NPC329077	246.22	2	7	4	122.82	0	1	0	0
NPC44530	302.28	0	6	5	110.38	0	0	0	0
NPC470802	426.50	6	6	5	110.38	0	0	0	0

In the process of drug discovery, a variety of physicochemical properties, collectively known as ADMET properties, play a crucial role in determining the likelihood of a compound entering clinical trials and ultimately becoming a successful drug. These properties encompass Molecular Weight (MW), which assesses a compound's size and its ability to cross biological membranes; Number of Rotatable Bonds (nRot), which evaluates a compound's flexibility and its susceptibility to unwanted transformations in the body; Number of Hydrogen Bond Acceptors (nHBacc), which gauges a compound's capacity to interact with and bind to proteins; Number of Hydrogen Bond Donors (nHBDon), which predicts a compound's potential to be metabolized by enzymes; and Topological Polar Surface Area (TPSA), which measures a compound's overall polarity and its ability to interface with water and other polar molecules. Generally, compounds with values within the following acceptable criteria are considered to have a higher likelihood of proving successful drug candidates: MW < 500 g/mol, nRot < 10, nHBacc < 10, nHBDon < 5, TPSA < 140 Å^2^. These criteria serve as valuable guidelines for early-stage drug discovery efforts, helping researchers identify compounds with favorable physicochemical properties that are more likely to progress through the drug development pipeline and ultimately reach patients.

Detailed ADMET analysis of top20 compounds are shown in Table 6. NPC105415 exhibits generally favourable ADME properties, with several desirable characteristics and minimal potential for adverse effects. It is not predicted to cause skin irritation or sensitization, and it exhibits a low likelihood of carcinogenicity and CYP enzyme inhibition. Additionally, its biodegradability and human intestinal absorption are both favorable. However, despite its overall positive profile, NPC105415 raises some concerns regarding its Ames mutagenicity, nephrotoxicity, reproductive toxicity, and plasma protein binding. These properties warrant further investigation to assess their potential impact on human health. NPC105415 exhibits a blend of favorable and potentially concerning ADME properties. While its low acute oral toxicity, low water solubility, and moderate plasma protein binding suggest a low likelihood of adverse effects, its Ames mutagenicity, nephrotoxicity, reproductive toxicity, and respiratory toxicity warrant further investigation. Additional studies are required to fully characterize NPC105415's ADME profile and assess its potential impact on human health. Contrary to the compound with the highest binding affinity in terms of molecular docking and MD simulation, further in-depth analysis of the ADME assessment of other compounds revealed that the other top compounds NPC117260 and NPC148409 possessed favorable properties in terms of Ames mutagenicity, nephrotoxicity, reproductive toxicity, and plasma protein binding. These findings suggest that the compound at the top of the list may have superior activity but may face challenges in advancing to the next phase due to ADME concerns, requiring optimization to enhance ADME properties.

**Table 6:** Comprehensive Assessment of ADME properties and toxicities of the best 20 natural products used in this study.

	NPC105415	NPC117260	NPC119767	NPC124300	NPC148409	NPC170742	NPC214729	NPC223136	NPC252590	NPC259098	NPC265454	NPC272174	NPC276930	NPC277205	NPC298778	NPC308931	NPC314573	NPC329077	NPC44530	NPC470802
Skin corrosion	−	−	−	−	−	−	−	−	−	−	−	−	−	−	−	−	−	−	−	−
Skin irritation	−	+	−	−	−	−	−	−	−	−	−	−	−	−	−	−	−	−	−	−
Skin sensitisation	−	+	−	−	−	−	−	−	−	−	−	−	−	−	−	−	−	−	−	−
Human oral bioavailability	−	−	−	−	−	−	−	−	−	+	−	+	−	−	−	−	−	−	−	−
Blood Brain Barrier	+	−	−	−	−	−	−	−	−	+	−	+	−	−	+	−	−	−	−	−
Biodegradation	−	−	−	−	−	−	−	−	−	−	−	−	−	−	−	−	−	−	−	−
Carcinogenicity (binary)	−	−	−	−	−	−	−	−	−	−	−	−	−	−	−	−	−	−	−	−
CYP1A2 inhibition	−	+	−	−	+	+	−	−	−	+	−	+	−	−	+	+	−	−	+	−
CYP2C9 inhibition	−	−	−	−	−	−	−	−	−	−	−	−	−	−	−	+	−	−	−	−
CYP2D6 inhibition	−	−	−	−	−	−	−	−	−	−	−	−	−	−	−	−	−	−	−	−
CYP inhibitory promiscuity	−	−	−	−	−	−	−	−	−	−	−	+	−	−	+	−	−	−	−	−
Hepatotoxicity	+	−	−	−	+	+	−	−	−	+	−	+	−	−	+	+	−	−	−	+
Human Intestinal Absorption	+	+	−	−	+	+	−	−	+	+	−	+	+	+	+	+	+	+	+	+
Ames mutagenesis	+	−	+	+	−	+	−	+	−	+	+	−	−	+	+	+	+	+	+	+
Nephrotoxicity	+	−	−	−	+	−	−	−	−	+	−	+	−	+	+	+	−	−	−	−
Reproductive toxicity	+	−	+	+	+	+	+	+	+	+	+	+	+	+	+	−	+	+	+	+
Acute Oral Toxicity	2.72	2.92	2.44	2.31	2.73	2.46	1.97	2.26	2.00	1.52	1.98	1.93	1.79	1.78	2.37	2.13	3.05	3.05	2.30	2.50
Plasma protein binding	1.06	0.78	0.75	0.79	0.88	1.08	0.55	0.73	0.59	1.01	0.73	0.91	0.75	0.65	0.92	1.11	1.07	1.07	0.93	0.94
Water solubility (logS)	− 3.17	− 1.96	− 2.32	− 2.45	− 3.35	− 2.95	− 2.45	− 2.45	− 2.62	− 2.71	− 2.45	− 2.71	− 2.90	− 3.16	− 4.24	− 3.18	− 3.26	− 3.26	− 3.96	− 2.66

**Figure 5:**
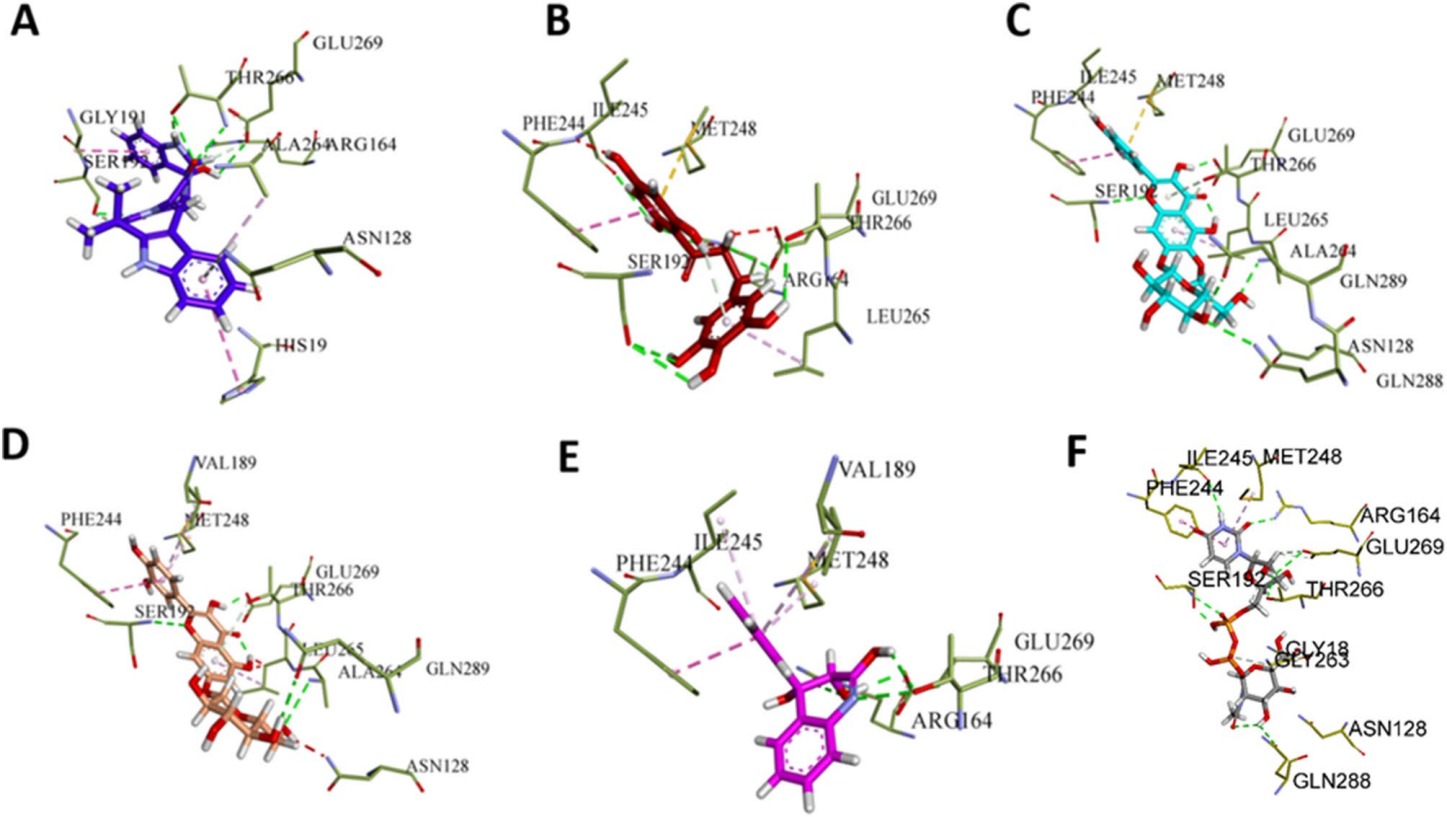
Docking structures of the best five NPs interacted with MurG binding site of *E. coli*. Ligands are presented in stick, while MurG residues are depicted in thin-stick with olive colour. The structures of A, B, C, D, E and F are NPC272174, NPC170742, NPC117260, NPC277205, NPC259098 NPs and UDP_GlcNAc, respectively. Important hydrogen bonds are highlighted.

The analysis of the binding modes for the top five natural compounds interacting with the MurG binding site was conducted to uncover their interactions. Notably, ARG164, SER192, THR266, and GLU269 emerged as crucial key residues involved in hydrogen bond interactions. Conversely, PHE244, LEU265 and MET248 amino acid residues are essential for hydrophobic interactions, namely pi-alkyl and pi-pi stacked interactions for all ligands except the top hit NPC272174, which involved in the hydrophobic interactions with surrounding amino acid residues HIS19, GLY191, SER192 and ALA264. Moreover, NPC170742 and NPC117260 ligands forms pi-sulfur interaction between MET248 and the aromatic ring of the ligand as shown in Fig. 5.

It's noteworthy that among all the binding site residues, Gln289, GLN288, ARG164, and GLU269 of *E. coli* MurG play a significant role in the interaction with UDP_GlcNAc substrate^46,87^. Additionally, observations indicate that the MurG active site features a conserved pocket as shown in Fig. 5F ^29,30^. This implies that the frameworks of the top five NPs form similar binding interactions as the UDP_GlcNAc substrate, as illustrated in Fig. 5. Comparing the interacting residues involved in binding interaction for the top five compounds, we observe some similarities and differences. Both NPC272174 and NPC170742 interact with ARG164, SER192, THR266, and GLU269 via hydrogen bonds, but NPC170742 also has additional interactions with ILE245 and a pi-sulfur bond with MET248. In contrast, NPC272174 has a pi-donor H-bond with ASN128 and π–π T-shaped interaction with HIS19. Both NPC117260 and NPC277205 interact with SER192, ALA264, GLU269, and THR266 (via pi-donor hydrogen bonds), as well as PHE244 via π–π stacked interactions, but NPC117260 also interacts with GLN288 and GLN289 via hydrogen bonds and MET248 via a π-sulfur bond. NPC277205 has an additional π–π stacked interaction with MET248 and π-alkyl interactions with LEU265. NPC259098 interacts with ARG164, THR266, and GLU269 via hydrogen bonds, and PHE244 via π–π stacked interactions, as well as π-alkyl contacts with ILE245 and MET248.

Although the binding scores for the top five compounds are relatively close, ranging from − 10.257 to − 9.818, the differences in their specific interactions with amino acid residues may account for differences in their overall binding and biological activities. The various types of interactions play significant roles in ligand–protein binding, contributing to the specificity and strength of the interaction. Hydrogen bonds, which are relatively common, help stabilize the complex and contribute to the specificity of the interaction. Π–π stacking contributes to the strength of the interaction and helps to orient the ligand in the binding site. π-alkyl interactions help increase the affinity of the ligand for the protein by reducing the overall energy of the complex. π-donor hydrogen bonds contribute to the specificity of the interaction and stabilize the complex, while π-sulfur interactions aid to orient the NPs in the binding site and contribute to the strength of the interaction.

### MD simulation analysis

Promising natural products (NPC727174, NPC170742, and NPC259098) and the standard UDP_GlcNAc stabilities within the active site of the MurG receptor were evaluated through a 100 ns MD simulation for each until the system reached convergence. The resulting total 400 ns trajectory was analyzed using root mean squared deviation (RMSD) to evaluate the dynamic behavior and stabilities of the standard and NPs-1nlm complexes, radius of gyration (Rg), root means square fluctuation (RMSF), transition path analysis, and free energy calculations. The “Supplementary video S1” provides details on the 100 ns MD simulations of the best-hit NPC272174 during the complexation with the MurG enzyme in *Escherichia coli*.

#### Root means squared deviation

**Figure 6:**
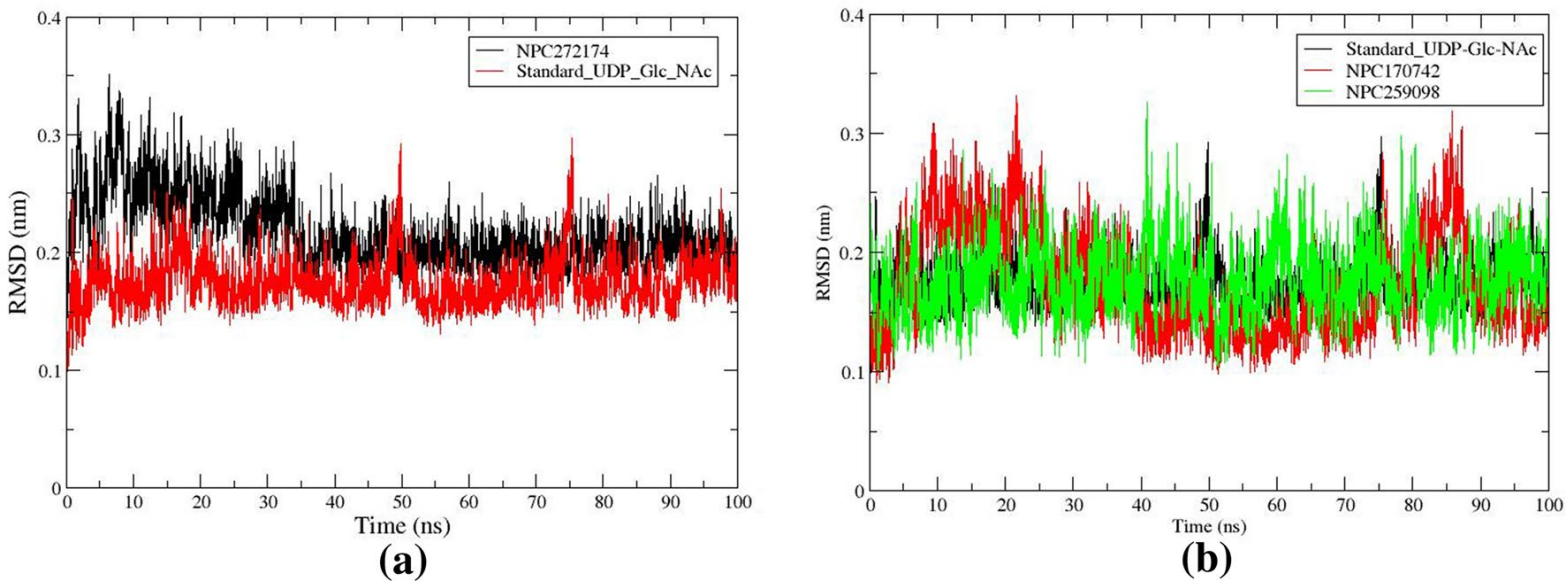
RMSD curve of the complexes of (**a**) NPC272174 and standard UDP_GlcNAc. (**b**) NPC170742, NPC259098, and standard UDP_GlcNAc backbone atoms complexed with MurG enzyme of *E. coli*.

Following the MD simulation, structural changes and stability were assessed by studying the root mean squared deviation from the start of the simulation runs and the initial conformational structures. RMSD calculates the deviation in the complex conformation compared to its initial conformation, providing insight into any changes in the structures during the complexation of ligands-MurG systems. Figure 6 illustrates the RMSD curve during the time for the (NPC727174, NPC170742, NPC259098, and standard UDP_GlcNAc) with MurG complexes. Throughout the experiment, it can be observed that the NPC727174-MurG complex undergoes a relative increase in RMSD values from the start of simulation to 0.35 nm within the first 10 ns as shown in Fig. 6a as compared to the standard UDP_GlcNAc. The high RMSD fluctuation from 0 to 30 ns appears to have happened due to the conformational change of the NPC272174 compound inside the MurG active site. Then, the plot began to stabilize after 30–100 ns. This result indicates that NPC272174 encourages the stability of the NPC272174-MurG complex. In addition, the NPC170742 complex behaves with the same increased fluctuation at the first 30 ns, Fig. 6b, while the NPC259098 complex behaves with constant fluctuations with an RMSD value of 0.2 nm. However, the overall RMSD for the NP complexes was consistently lower over the simulation time, indicative of the fact that the complexes after 30 ns simulation time remained largely unchanged from the original input structure and were reasonably stable and capable of maintaining its conformation, specifically the best-hit NPC727174-MurG complex structure.

#### Radius of gyration

**Figure 7:**
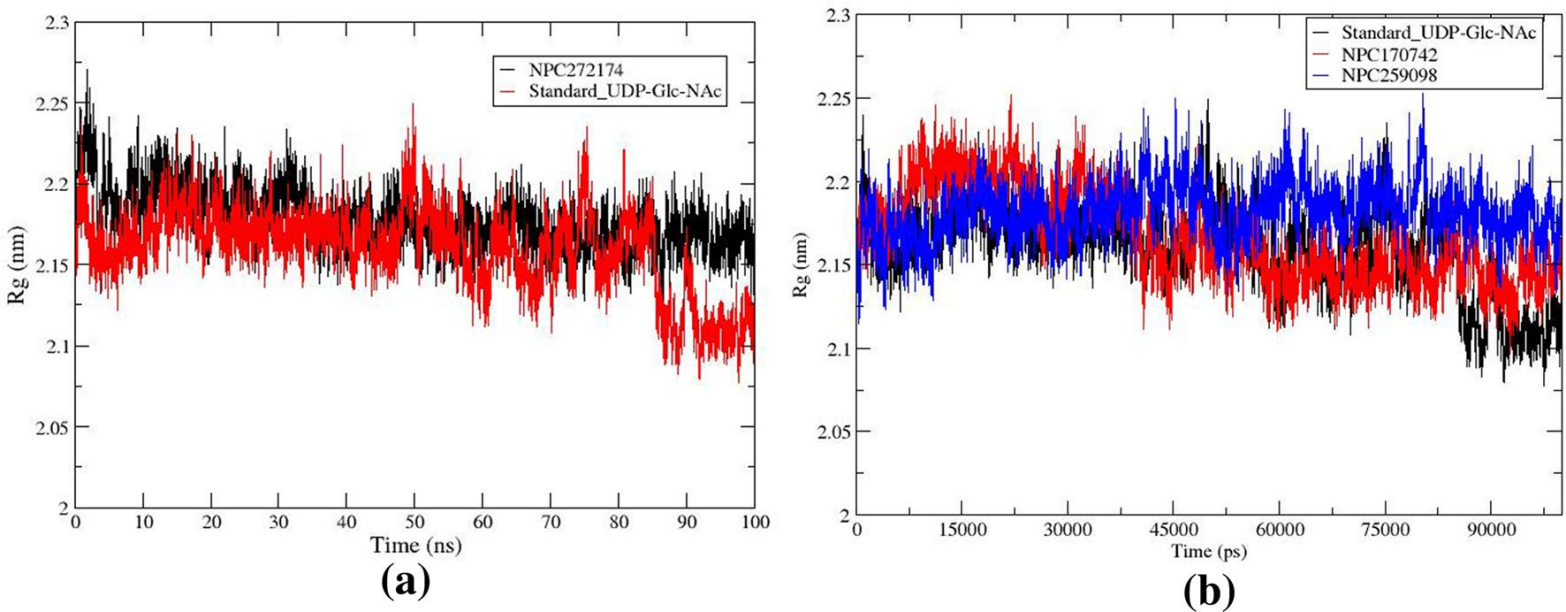
Rg profile of the compounds (**a**) NPC272174 and standard UDP_GlcNAc. (**b**) NPC170742, NPC259098, and standard UDP_GlcNAc complexed with MurG enzyme of *E. coli*.

The radius of gyration (Rg) serves as an indicator of the folding stability of the MurG receptor during the interaction of NP compounds. High folded enzyme conformation is reflected in greater compactness, resulting in a lower Rg value. Conversely, an increasing Rg value suggests less compactness and indicates an unfolded structure. In the present study, Rg was employed to assess the compactness of the systems throughout the simulations, and the Rg plots are shown in Fig. 7. The findings reveal Rg scores ranging between 2.15 and 2.25 nm for NPC727174-MurG complex, Fig. 7a. The reported Rg value for the MD simulation was 2.17 nm over the simulation time. While minor flexibility was observed at the beginning of the MD simulation, the values quickly became constant and fluctuated within a permitted range after 30 ns. NPC170742 and NPC259098 complexes behaved as NPC727174 complex with Rg scores ranging between 2.12 and 2.24 nm as shown in Fig. 7b.

#### Root mean squared fluctuations

**Figure 8:**
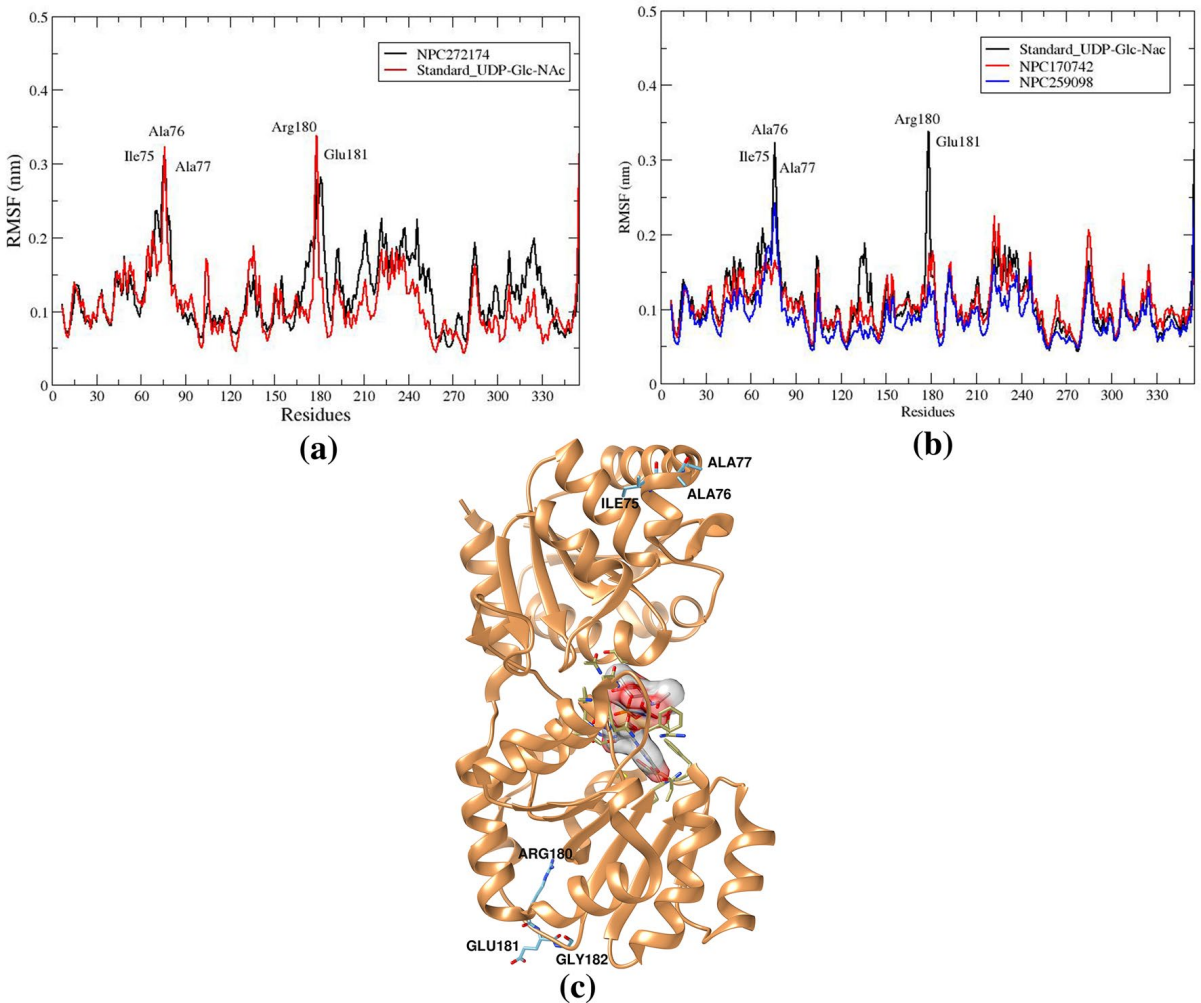
Fluctuation profile (**a**) RMSF plot of the NPC272174 and standard UDP_GlcNAc compounds. (**b**) RMSF plot of the NPC170742, NPC259098, and standard UDP_GlcNAc compounds complexed with MurG enzyme during the simulation time. (**c**) Amino acid residues involve the highest RMSF value in the ligands-MurG complex structures.

To describe the fluctuations and structural changes within the NP-MurG complexes, root mean square fluctuations (RMSF) were studied and the results are depicted in Fig. 8. Investigation of the RMSFs indicates that the most elevated fluctuation, approximately 0.30 nm, occurred consistently throughout the simulation period for NPC727174 and the standard UDP_GlcNAc. This high fluctuation was associated with amino acid residues ALA76, Ile75, ALA77, Arg180, and GLU181, Fig. 8a, which were observed in the N-terminal region and the loop in the carboxy-terminal side as shown in Fig. 8c. In addition, it has been observed that the NPs NPC170742 and NPC259098 have the same fluctuation regions in the MurG enzyme. This observation agrees with a prior study including the crystal of MurG, which identified flexible areas in the carboxy-terminal and N-terminal amino acid residues^31,87^.

#### Free energy analysis

The MM-PBSA method has been applied to predict binding free energy and to evaluate the relative stability of the NPC727174, NPC170742, NPC259098, and the standard UDP_GlcNAc complexed with the MurG enzyme. The calculated total binding energy for MD trajectories and the obtained binding free energy components are shown in Table 7. As can be seen, the calculated free energies, ΔG_Total_, of MurG enzyme towards the different NPs and the standard are in the order of NPC259098, − 15.64 kJ mol^−1^ > NPC170742, − 20.72 kJ mol^−1^ > NPC727174, − 33.64 kJ mol^−1^ > UDP_GlcNAc, − 40.57 kJ mol^−1^.

**Table 7:** Calculated binding free energies (in kJ mol^−1^) and their components based on the MM-GBSA method for the three MurG-NPs complexes and the standard UDP_GlcNAc-MurG complex.

Energy components	NPC727174	NPC170742	NPC259098	UDP_GlcNAc
van der Waals (ΔE_vdW_)	− 21.31	− 21.98	− 26.19	− 51.68
Electrostatic (ΔE_ele_)	− 60.88	− 67.51	− 7.8	− 71.39
Polar solvation (ΔE_PSE_)	56.40	72.78	21.37	89.77
SASA (ΔES_SASA_)	− 3.88	− 4.01	− 3.01	− 7.27
Total binding energy (ΔG_Total_)	− 33.64	− 20.72	− 15.64	− 40.57

From the contribution of the calculated energy components of the binding free energies in the NPC727174, NPC259098, and the standard UDP_GlcNAc as shown in Table 7, the main driving force for the binding interactions is electrostatic and van der Waals interactions. The polar solvation contributed unfavourably to the binding of the ligand to MurG enzyme. Indeed, the electrostatic, van der Waals non polar, and SASA interactions contribute favourably towards the binding of all ligands to MurG enzyme and are compensated by the large polar solvation energy.

**Figure 9:**
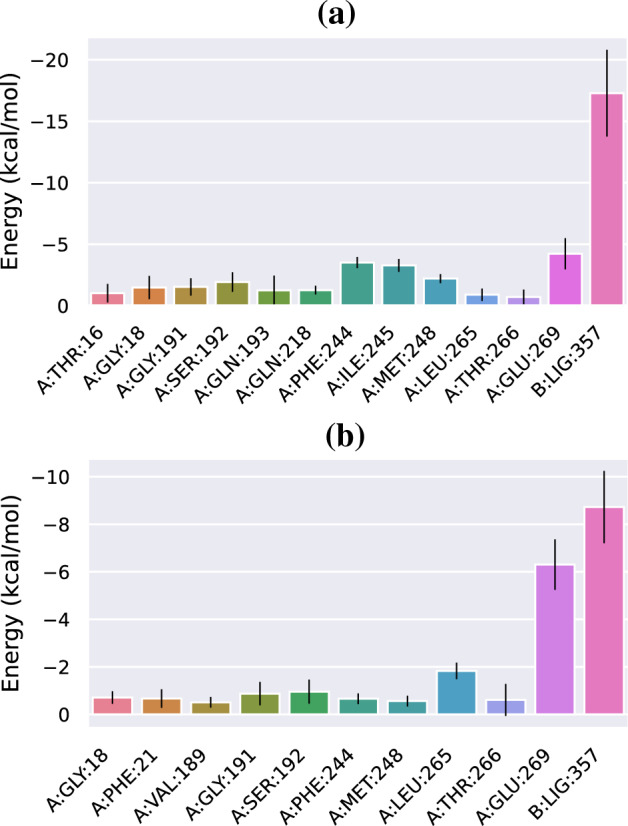
Energetic components per-residue decomposition of (**a**) the standard UDP_GlcNAc. (**b**) NPC727174 complexes with MurG enzyme of *E. coli*.

A detailed analysis of the binding energy contributions was analysed using the MM-PBSA method. Figure 9 shows the energy contributions in kcal mol^−1^ of the ligand–receptor per-residue interaction for the standard UDP_GlcNAc and NPC727174 ligand systems. During the simulation of the standard UDP_GlcNAc complex, the energy appears mainly from the binding with the polar Glu269 amino acid residue and nonpolar Phe244, Ile245 and Met248 amino acid residues, Fig. 9a. On the other hand, the nonpolar amino acid residue of the NPC727174 complex interactions mainly arose from Phe21, Phe244 and Met248 Leu265, with the strongest interactions from Leu265 amino acid residue as shown in Fig. 9b. In addition, amino acid residues Glu269 made obvious polar contributions to NPC727174 with lowest binding energy value and strongest interaction as compared to the standard UDP_GlcNAc complex. This indicates that the polar amino acid residue interaction stabilizes the NPC727174-MurG interaction during the simulation. While the stability of the UDP_GlcNAc ligand in the MurG active site is achieved via hydrophobic and polar interactions.

#### Analysis of the complex transition path

**Figure 10:**
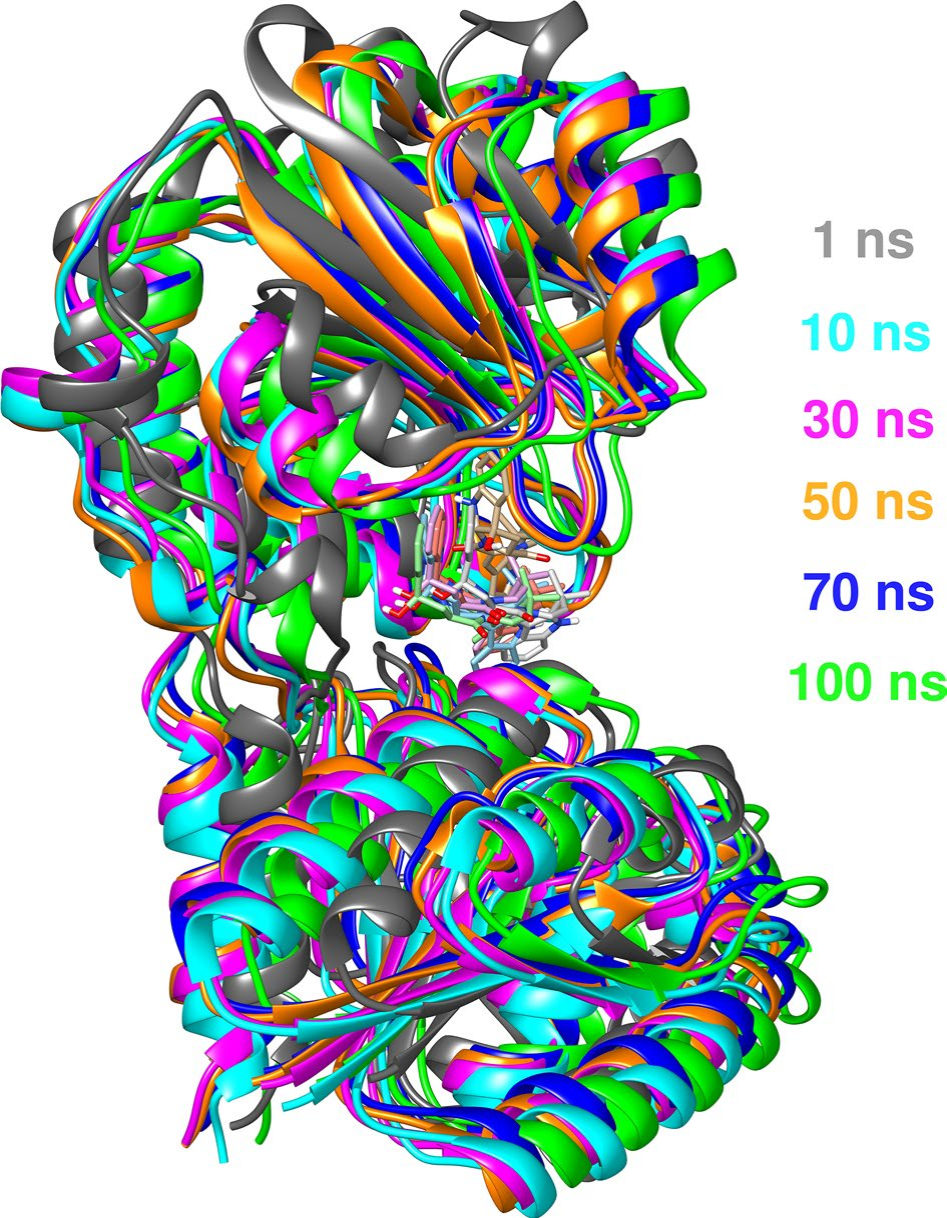
Overlays of the conformational dynamics snapshot for NPC272174-MurG complex taken at different simulation times. The conformation colors corresponding to the appropriate time are highlighted.

The motions of NPC272174 and MurG enzyme complex at different simulation periods were investigated and shown in Fig. 10. The most changes involve the coil of the C-terminal and hinge regions which move fast at the start of the simulation time, and this agrees with the increased fluctuation results obtained from the RMSD plot at the first 30 ns, then persistent value was observed for the remaining simulation time till 100 ns. The transition path results indicate that NPC272174 natural compound enhances the complex stability along the simulation time.

## Conclusion

Many studies identified inhibitors for the antibiotic target MurG enzyme. However, the nucleus structure for large MurG enzyme inhibitors is restricted to peptidoglycan-mimicking scaffolds, thus simulating the resistance of drugs in the *E. coli* strains. Consequently, the development of new MurG potential inhibitors has increased to a favorable approach. We analyzed the best five hits from the NPASS database of natural products as potential MurG inhibitors, followed by the drug-likeness assessment using a computational prediction method. The results indicated that the promising NPs bind with an increased affinity, mimicking the binding of the UDP_GlcNAc substrate. The best five hits form H-bond interaction with important amino acid residues of ARG164, SER192, THR266, and GLU269. A molecular dynamics simulation study of the best three candidate natural compounds complexed with MurG demonstrated strong stability of NPC272174 to the enzyme structure. In addition, upon comparing the MM-PBSA binding free energy values for the three NPs, the results suggest that the complexation of NPC272174 to the MurG is more favorable. Therefore, the identified NPs suggest that the potential NPC272174 compound is a promising novel scaffold inhibitor for the MurG protein in *E. coli* as an antibacterial agent.

### Supplementary Information

Supplementary Table.

Supplementary Video 1.

## Data Availability

All data generated or analysed during this study are included in this published article [and its supplementary information files].
