# Supplementary material for: A comprehensive computational study to explore promising natural bioactive compounds targeting glycosyltransferase MurG in Escherichia coli for potential drug development
Source: Sci Rep. 2024 Mar 26;14:7098. doi: 10.1038/s41598-024-57702-x (PMC10966019; doi:10.1038/s41598-024-57702-x)
Supplement: Supplementary file 1 — Supplementary Table. [file 41598_2024_57702_MOESM1_ESM.docx]

**Supporting Information (SI)**

A comprehensive computational study to explore promising natural bioactive compounds targeting glycosyltransferase MurG in *Escherichia coli* for potential drug development

**Amneh Shtaiwi ^*1^, Shafi Ullah Khan ^2,3^, Meriem Khedraoui ^4^, Mohd Alaraj ^5^, Abdelouahid Samadi ^*6^, Samir Chtita ^4^**

^1^ Faculty of Pharmacy, Middle East University, Queen Alia Airport Street, 11610 Amman, Jordan. Email: [ashtaiwi@meu.edu.jo](mailto:ashtaiwi@meu.edu.jo); Tel: +96264790222; [ORCID: 0000-0001-6081-7440](https://orcid.org/0000-0001-6081-7440)

^2^ Interdisciplinary Research Unit for Cancer Prevention and Treatment, Baclesse Cancer Centre, Université de Caen Normandie Inserm Anticipe UMR 1086, Normandie Univ, Research Building, F‑14000 François 3 Avenue Général Harris, BP 45026, 14 076, cedex 05 Caen, France. Email: [shafi-ullah.khan@unicaen.fr](mailto:shafi-ullah.khan@unicaen.fr); ORCID: 0000-0001-9231-1831

^3^ Centre François Baclesse, Avenue Général Harris, 14076, Caen Cedex, France. Email: [shafi-ullah.khan@unicaen.fr](mailto:shafi-ullah.khan@unicaen.fr)

^4^ Laboratory of Analytical and Molecular Chemistry, Faculty of Sciences Ben M’Sik, Hassan II University of Casablanca, B. P 7955 Casablanca, Morocco. Email: [samirchtita@gmail.com](mailto:samirchtita@gmail.com); ORCID: 0000-0003-2344-5101

^5^ Faculty of Pharmacy, University of Jerash, Jerash, Jordan. Email: [m.araj@jpu.edu.jo](mailto:m.araj@jpu.edu.jo)

^6^ Department of Chemistry, College of Science, UAEU, P.O. Box No. 15551, Al Ain, UAE. Email: [samadi@uaeu.ac.ae](mailto:samadi@uaeu.ac.ae)

*Corresponding author:

Amneh Shtaiwi ([ashtaiwi@meu.edu.jo](mailto:ashtaiwi@meu.edu.jo)) & Abdelouahid Samadi ([samadi@uaeu.ac.ae](mailto:samadi@uaeu.ac.ae))

**Supplementary video S1:** 100 ns MD simulations of the best hit NPC272174 during the complexation with the MurG enzyme in *Escherichia coli*.

**Supplementary Python script S1:** Detailed average binding energy calculation python script "MmPbSaStat.py" is provided in the following *g_mmpbsa* link:

<https://raw.githubusercontent.com/RashmiKumari/g_mmpbsa/master/tools/MmPbSaStat.py>

**Supplementary Python script S2:** Detailed contribution of residues to the binding energy python script "MmPbSaDecomp.py" is provided in the following *g_mmpbsa* link:

<https://raw.githubusercontent.com/RashmiKumari/g_mmpbsa/master/tools/MmPbSaDecomp.py>

**Table S1.** Detailed binding interactions of top 5 Hits with the amino acid residue of targeted protein

| Distance | Category | Types | From | From Chemistry | To | To Chemistry |
| --- | --- | --- | --- | --- | --- | --- |
| 1st | | | | | | |
| 3.33675 | Hydrogen Bond | Conventional Hydrogen Bond | A:ARG164:NH1 | H-Donor | :NPC272174:O34 | H-Acceptor |
| 2.92444 | Hydrogen Bond | Conventional Hydrogen Bond | A:SER192:OG | H-Donor | :NPC272174:O33 | H-Acceptor |
| 2.50301 | Hydrogen Bond | Conventional Hydrogen Bond | A:THR266:N | H-Donor | :NPC272174:O32 | H-Acceptor |
| 2.9669 | Hydrogen Bond | Conventional Hydrogen Bond | A:THR266:OG1 | H-Donor | :NPC272174:O32 | H-Acceptor |
| 1.63774 | Hydrogen Bond | Conventional Hydrogen Bond | :NPC272174:H57 | H-Donor | A:THR266:OG1 | H-Acceptor |
| 2.75188 | Hydrogen Bond | Conventional Hydrogen Bond | :NPC272174:H58 | H-Donor | A:GLU269:OE1 | H-Acceptor |
| 2.73449 | Hydrogen Bond | Carbon Hydrogen Bond | :NPC272174:H45 | H-Donor | A:SER192:OG | H-Acceptor |
| 2.92846 | Hydrogen Bond | Carbon Hydrogen Bond | :NPC272174:H49 | H-Donor | A:GLU269:OE1 | H-Acceptor |
| 3.53613 | Hydrogen Bond | Pi-Donor Hydrogen Bond | A:ASN128:ND2 | H-Donor | :NPC272174 | Pi-Orbitals |
| 5.58834 | Hydrophobic | Pi-Pi T-shaped | A:HIS19 | Pi-Orbitals | :NPC272174 | Pi-Orbitals |
| 4.39993 | Hydrophobic | Amide-Pi Stacked | A:GLY191:C,O;SER192:N | Amide | :NPC272174 | Pi-Orbitals |
| 4.79462 | Hydrophobic | Pi-Alkyl | :NPC272174 | Pi-Orbitals | A:ALA264 | Alkyl |
| 2nd | | | | | | |
| 2.88411 | Hydrogen Bond | Conventional Hydrogen Bond | A:ARG164:NH1 | H-Donor | :NPC170742:O21 | H-Acceptor |
| 3.13 | Hydrogen Bond | Conventional Hydrogen Bond | A:SER192:OG | H-Donor | :NPC170742:O18 | H-Acceptor |
| 2.7517 | Hydrogen Bond | Conventional Hydrogen Bond | :NPC170742:H31 | H-Donor | A:THR266:OG1 | H-Acceptor |
| 2.88127 | Hydrogen Bond | Conventional Hydrogen Bond | :NPC170742:H35 | H-Donor | A:ILE245:O | H-Acceptor |
| 2.99384 | Hydrogen Bond | Conventional Hydrogen Bond | :NPC170742:H36 | H-Donor | A:SER192:OG | H-Acceptor |
| 2.14834 | Hydrogen Bond | Conventional Hydrogen Bond | :NPC170742:H37 | H-Donor | A:GLU269:OE1 | H-Acceptor |
| 2.79408 | Hydrogen Bond | Conventional Hydrogen Bond | :NPC170742:H38 | H-Donor | A:GLU269:OE1 | H-Acceptor |
| 2.6917 | Hydrogen Bond | Carbon Hydrogen Bond | :NPC170742:H29 | H-Donor | A:GLU269:OE1 | H-Acceptor |
| 3.04892 | Hydrogen Bond | Pi-Donor Hydrogen Bond | :NPC170742:H34 | H-Donor | :NPC170742 | Pi-Orbitals |
| 3.80049 | Other | Pi-Sulfur | A:MET248:SD | Sulfur | :NPC170742 | Pi-Orbitals |
| 4.30998 | Hydrophobic | Pi-Pi Stacked | A:PHE244 | Pi-Orbitals | :NPC170742 | Pi-Orbitals |
| 5.17925 | Hydrophobic | Pi-Alkyl | :NPC170742 | Pi-Orbitals | A:LEU265 | Alkyl |
| 3rd | | | | | | |
| 3.26962 | Hydrogen Bond | Conventional Hydrogen Bond | A:SER192:N | H-Donor | :NPC117260:O24 | H-Acceptor |
| 2.29616 | Hydrogen Bond | Conventional Hydrogen Bond | A:ALA264:N | H-Donor | :NPC117260:O33 | H-Acceptor |
| 2.79044 | Hydrogen Bond | Conventional Hydrogen Bond | A:GLN288:NE2 | H-Donor | :NPC117260:O31 | H-Acceptor |
| 1.76754 | Hydrogen Bond | Conventional Hydrogen Bond | :NPC117260:H52 | H-Donor | :NPC117260:O23 | H-Acceptor |
| 1.71445 | Hydrogen Bond | Conventional Hydrogen Bond | :NPC117260:H53 | H-Donor | A:GLU269:OE2 | H-Acceptor |
| 2.0201 | Hydrogen Bond | Conventional Hydrogen Bond | :NPC117260:H55 | H-Donor | A:GLN289:OE1 | H-Acceptor |
| 3.74794 | Hydrogen Bond | Pi-Donor Hydrogen Bond | A:THR266:OG1 | H-Donor | :NPC117260 | Pi-Orbitals |
| 3.32332 | Hydrogen Bond | Pi-Donor Hydrogen Bond | A:THR266:OG1 | H-Donor | :NPC117260 | Pi-Orbitals |
| 3.80546 | Other | Pi-Sulfur | A:MET248:SD | Sulfur | :NPC117260 | Pi-Orbitals |
| 4.51718 | Hydrophobic | Pi-Pi Stacked | A:PHE244 | Pi-Orbitals | :NPC117260 | Pi-Orbitals |
| 5.18186 | Hydrophobic | Pi-Alkyl | :NPC117260 | Pi-Orbitals | A:LEU265 | Alkyl |
| 4th | | | | | | |
| 3.08291 | Hydrogen Bond | Conventional Hydrogen Bond | A:SER192:N | H-Donor | :NPC277205:O23 | H-Acceptor |
| 3.06176 | Hydrogen Bond | Conventional Hydrogen Bond | A:ALA264:N | H-Donor | :NPC277205:O32 | H-Acceptor |
| 1.78714 | Hydrogen Bond | Conventional Hydrogen Bond | :NPC277205:H48 | H-Donor | :NPC277205:O22 | H-Acceptor |
| 1.98636 | Hydrogen Bond | Conventional Hydrogen Bond | :NPC277205:H49 | H-Donor | A:GLU269:OE2 | H-Acceptor |
| 2.26547 | Hydrogen Bond | Conventional Hydrogen Bond | :NPC277205:H51 | H-Donor | A:GLN289:OE1 | H-Acceptor |
| 4.03403 | Hydrogen Bond | Pi-Donor Hydrogen Bond | A:THR266:OG1 | H-Donor | :NPC277205 | Pi-Orbitals |
| 4.6995 | Hydrophobic | Pi-Pi Stacked | A:PHE244 | Pi-Orbitals | :NPC277205 | Pi-Orbitals |
| 5.4425 | Hydrophobic | Pi-Alkyl | :NPC277205 | Pi-Orbitals | A:VAL189 | Alkyl |
| 4.52914 | Hydrophobic | Pi-Alkyl | :NPC277205 | Pi-Orbitals | A:MET248 | Alkyl |
| 5.28024 | Hydrophobic | Pi-Alkyl | :NPC277205 | Pi-Orbitals | A:LEU265 | Alkyl |
| 5th | | | | | | |
| 2.86785 | Hydrogen Bond | Conventional Hydrogen Bond | A:ARG164:NH1 | H-Donor | :NPC259098:O18 | H-Acceptor |
| 2.80274 | Hydrogen Bond | Conventional Hydrogen Bond | A:ARG164:NH1 | H-Donor | :NPC259098:O19 | H-Acceptor |
| 2.69996 | Hydrogen Bond | Conventional Hydrogen Bond | A:THR266:OG1 | H-Donor | :NPC259098:N16 | H-Acceptor |
| 2.33985 | Hydrogen Bond | Conventional Hydrogen Bond | :NPC259098:H30 | H-Donor | A:THR266:OG1 | H-Acceptor |
| 1.93808 | Hydrogen Bond | Conventional Hydrogen Bond | :NPC259098:H31 | H-Donor | A:GLU269:OE2 | H-Acceptor |
| 4.2067 | Hydrophobic | Pi-Pi Stacked | A:PHE244 | Pi-Orbitals | :NPC259098 | Pi-Orbitals |
| 5.36755 | Hydrophobic | Pi-Alkyl | :NPC259098 | Pi-Orbitals | A:VAL189 | Alkyl |
| 5.47954 | Hydrophobic | Pi-Alkyl | :NPC259098 | Pi-Orbitals | A:ILE245 | Alkyl |
| 4.32099 | Hydrophobic | Pi-Alkyl | :NPC259098 | Pi-Orbitals | A:MET248 | Alkyl |
